# Neocortical Lamination: Insights from Neuron Types and Evolutionary Precursors

**DOI:** 10.3389/fnana.2017.00100

**Published:** 2017-11-07

**Authors:** Gordon M. Shepherd, Timothy B. Rowe

**Affiliations:** ^1^Department of Neuroscience, Yale University School of Medicine, New Haven, CT, United States; ^2^Jackson School of Geosciences, University of Texas at Austin, Austin, TX, United States

**Keywords:** amniote, intratelencephalic, serial homology, cortical hierarchy, olfactory cortex, feedback excitation, feedback inhibition, gene expression

## Abstract

The neocortex is characterized by lamination of its neuron cell bodies in six layers, but there are few clues as to how this comes about and what is its function. Recent studies provide evidence that evolution from simple three-layer cortex may give insight into this problem. Three-layer cortex arose in the olfactory, hippocampal and dorsal cortex of the early amniote forebrain based on a cortical module of excitatory and inhibitory inputs to an intratelencephalic (IT) type of pyramidal neuron with feedback excitation and inhibition and related interneurons. We summarize recent evidence suggesting the hypothesis that the developmental program of three-layer olfactory cortex was co-opted to form six-layer mammalian neocortex, elaborating IT cortical units in layers 2–6 while adding layer 4 stellate cells, layer 5B pyramidal tract (PT) cells and layer 6 corticothalamic (CT) cells.

Initial evidence for cortical function at the cellular level came from stained cell bodies in mammals, which were found to be arranged in six layers that varied in different cortical areas. Similarity of the layers in different areas suggested a similarity of functions, whereas differences suggested different properties related to different cortical systems. A century of research on lamination produced a useful guide to cell location and function. However, cortical layering is variable among amniotes (Mammalia plus Reptilia, including Aves), and is absent in birds (Streidter, 2005). Both six-layer mammalian cortex and the absence of cortical layering in birds are now understood to have evolved from a common ancestor inferred to possess three cortical layers (Ulinski, 1983; ten Donkelaar, 1998; Rowe and Shepherd, 2016; Rowe, 2017). This variation raises the question of a more general cortical organization, one based not on lamination but on fundamental neuron functions, dependent on dendrites, axons, synapses and their physiological properties, connections and actions. Can a more general cortical organization be inferred in amniotes ancestrally, one which underlies both layering and its loss in descendent lineages?

Methods from gene targeting to physiological and pharmacological analysis are emerging to decipher neuron functions across different cortical areas. A synthesis of this work (Harris and Shepherd, 2015) identified a rich domain of inquiry in patterns of connectivity, the hodology, between genetically defined cell types as a primary organizing principle of the neocortex. Parallel work sets the functional organization of cortical neurons within an explicit evolutionary context. These suggest the existence of a basic cortical circuit that had its origin in three-layer forebrain cortex of the ancestral amniote, that was conserved in non-avian reptiles, and that became elaborated in mammalian six-layer neocortex (Shepherd, 2011; Rowe and Shepherd, 2016).

We summarize these approaches to suggest a new synthesis of the evolution of neocortical neuron types. It offers insights into the ancestral amniote three-layer cortex as an associative network of higher level functions. It presents the mammalian neocortex as a further elaboration of this network that came to directly influence the entire neuraxis as it further elaborated higher functions including multidimensional perception, memory, planning and execution. This emphasis on fundamental neuron functions and general cortical organization adds new insight into the roles of gene duplications, olfaction, somatosensation and motor control in driving neocortical evolution.

## Neocortical Cell Types and Their Laminar Location

**Figure 1:**
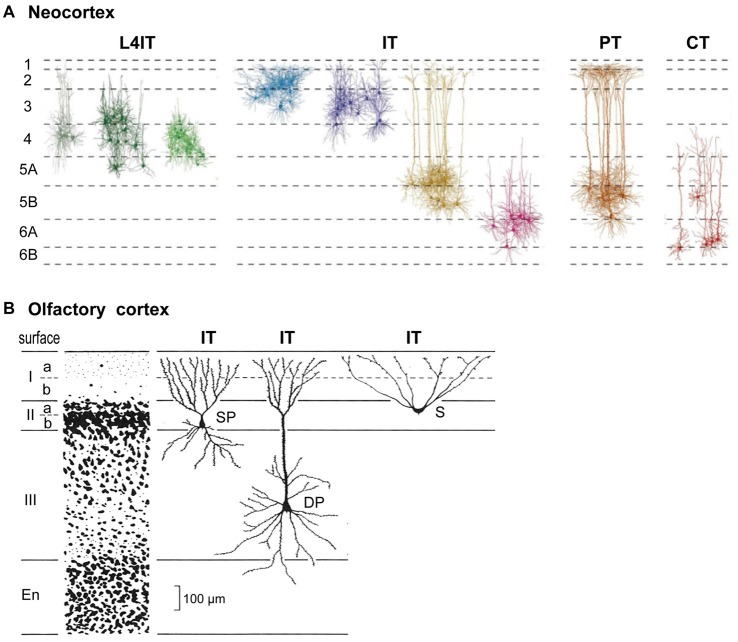
Basic cortical neuron types and laminae. **(A)** Neocortex (S1 barrel cortex). Intratelencephalic (IT) cells are found in layers 2–6; they connect only within the neocortex and basal ganglia. Pyramidal tract (PT) cells are restricted to L5B; they connect through the PT to the entire neuraxis. Corticothalamic (CT) cells are restricted to L6; they connect to the thalamus. L4IT: found mostly in L4, connect locally. See text. Adapted from Oberlaender et al. (2012) and Harris and Shepherd (2015). **(B)** Olfactory cortex IT pyramidal neurons are found in layers IIa, IIb and III. S, semilunar cells (lack basal dendrites); SP, small pyramids; DP, deep pyramids. Adapted from Neville and Haberly (2004).

Connectivity of pyramidal cells (PCs) in different neocortical laminae is summarized in Figure 1. Complementary to classifying cells in terms of their layers or morphology, pyramidal neurons carrying cortical output can be divided into four main types based on their output targets (Reiner et al.’s, 1998; Oberlaender et al., 2012; Harris and Shepherd, 2015).

A key type is the intratelencephalic (IT) pyramidal neuron, located in all layers from L2-L6, whose axon projects within the telencephalon and to other cortical areas or to the striatum. There are different combinations of inputs in each layer; the outputs are also diverse and exceed what can be covered here (for a summary, see Table 1, Harris and Shepherd, 2015). IT cells are excitatory, as are all cortical PCs. IT neurons are highly diverse, yet their connectivity patterns appear to be similar in different cortical areas. We will see that this is evidence of their evolutionary origin.

The outcome of physiological activity in IT cells must be carried to the rest of the nervous system. In basal amniotes (inferred from comparing living turtles, lizards and amphibians), this depended on connections from basal ganglia to neurons in the midbrain and brainstem which relayed the outcome to the rest of the neuraxis. By contrast, the mammalian neocortex evolved its own output neuron in the form of a cell that sends its long axon from the cortex through the pyramidal tract (PT) to the brain stem and spinal cord, carrying cortical output to many centers and nuclei. This PT cell is found in a specific sublayer, L5B. The long apical dendrite of PT cells extends toward L1, receiving inputs from every intervening layer; on the output side, subsets of PT neurons innervate different combinations of targets within the brainstem and spinal cord. These complex combinations of targets mirror the complex combinations of cortical neurons and their inputs for which PT cells are the final common path.

The third main type with a long axon is the corticothalamic (CT) cell. Its cell body is found in a single sublayer, L6, distinct from IT cells also found there. Its axon projects to the thalamic nucleus related to the area in which it is located. In primary sensory areas this goes to the specific sensory relay nucleus and associated reticular nucleus. In motor and association areas the thalamic relations are less clear. Traditionally it has been recognized that CT cells are part of a loop with thalamocortical (TC) cells binding the cortex and thalamus.

A final cortical cell type is the short-axon IT cell (Figure 1), localized mostly in layer 4 (L4); it receives thalamic inputs and sends its axon only locally. In sensory areas the L4IT cell usually lacks an apical dendrite, appearing as a stellate cell; elsewhere it is often a pyramidal or other type. Its axon usually targets only nearby cells in L2/3 or L5.

Thus, the main interaction between cortical areas is through the IT cells, and can be subsumed as “serial homology” (Harris and Shepherd, 2015), the idea that cell types and connections did not evolve independently in each area, but rather that a general, repeated organization was adapted for different functions in different locales. Another principle is the concept of “cortical hierarchy”, for example, between primary and secondary sensory areas. In some cases this involves outputs and inputs involving different layers, but this is not a universal rule. There may be a limited number of IT subclasses that are homologous in the sense that they arose at more general levels of the hierarchy and connect different cortical areas.

Three main types of cortical interneurons have emerged: parvalbumin (Pvalb), somatostatin (Sst) and vasoactive intestinal peptide (Vip), all inhibitory, but with specific connections with the PCs and with each other. Serial homologous principles appear to apply to their interactions in different areas, but are beyond the focus of this review.

## Evolutionary Origins of Neocortical Neurons and Lamination

Cortical lamination is not unique to neocortex. From an evolutionary perspective it began with simple three-layer cortex. The basic circuit organization within a three-layered cortical structure was established by Haberly’s classic study of olfactory cortex (summarized in Neville and Haberly, 2004), and later extended to general circuit organization in dorsal cortex and hippocampus in all non-avian reptiles (Güntürkün et al., 2017; Naumann and Laurent, 2017). Olfactory cortex in the ancestral amniote is inferred to have contained three subtypes of pyramidal neuron—semilunar, superficial and deep pyramidal—with distinct morphologies, located at successive depths that define specific cell sublayers (Figure 1B). This may indicate an underlying potential for three-layer cortex to evolve sublayers related to different pyramidal neurons at different depths, as eventually expressed most highly in neocortex. The three subtypes develop in an inside-out sequence, the same sequence expressed in neocortex (Luzzati, 2015; Klingler, 2017).

Our focus on a physiological approach to neocortex began with the synaptic organization of the mammalian olfactory cortex as a simple system for elucidating organizational principles. This suggested a basic circuit of PC with feedforward and feedback excitation and inhibition as a foundation for all cortical structures (Haberly and Shepherd, 1973; Shepherd, 1974). This was followed by the proposal that olfactory cortex is not a low level processing station, but rather is a high level association cortex that implements content addressable memory (Haberly, 1985). This critical insight underlies a growing consensus that higher association functions were already built into simple three layer cortex.

The functional organization of turtle dorsal cortex put these common features in a comparative evolutionary context (Smith et al., 1980; Kriegstein and Connors, 1986) in which six-layer mammalian neocortex evolved from a three-layer cortex in the ancestral amniote (Shepherd, 1988, 2011; Rowe and Shepherd, 2016). This has become an active field, with many current studies testing these ideas (Aboitiz and Montiel, 2015; Brunjes and Osterberg, 2015; Fournier et al., 2015; Luzzati, 2015; Naumann et al., 2015; Diodato et al., 2016; Klingler, 2017).

**Figure 2:**
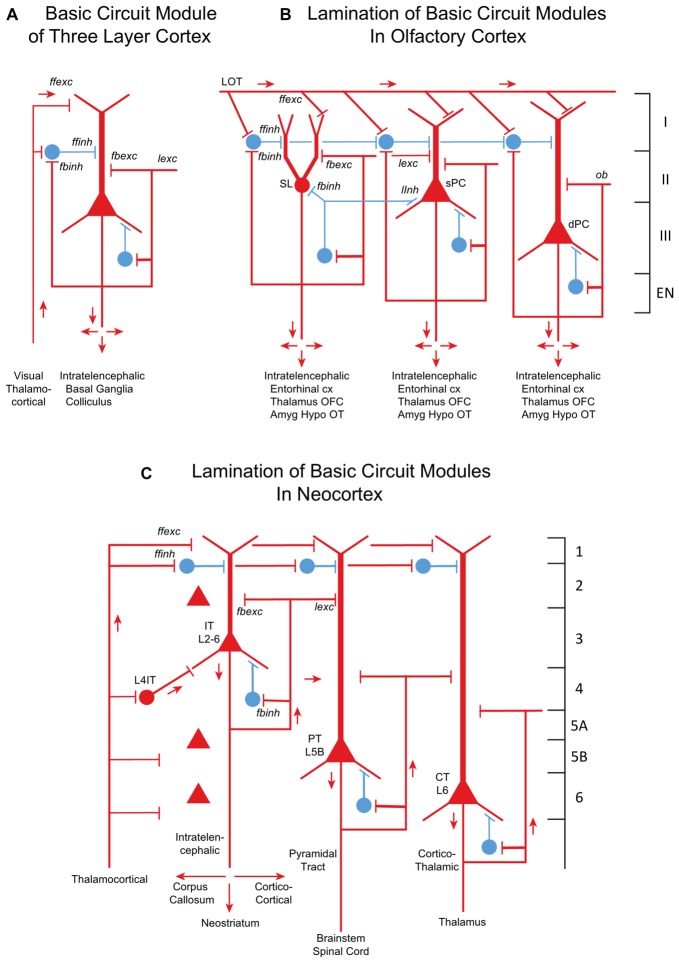
From three-layer to six-layer cortical microcircuits. **(A)** Simplified basic cortical module of ancestral three-layer olfactory cortex, hippocampus and reptilian dorsal cortex. Based on Kriegstein and Connors (1986) and Shepherd (2011). PC, pyramidal cell. Abbreviations of functional actions: ffexc, feedforward excitation; ffinh, feedforward inhibition; fbexc, feedback excitation; fbinh, feedback inhibition; lexc, lateral excitation; linh, lateral inhibition. **(B)** Olfactory cortex: lamination of basic circuit modules. **(C)** Mammalian neocortex: lamination of basic circuit modules. Abbreviations as in **(A)**. Laminae for the cell types are indicated. Presumed excitatory cells shown in red, inhibitory cells shown in blue. Based on Shepherd (1988) and Rowe and Shepherd (2016).

The core of the amniote cortex is a layer of PCs with long apical dendrites (Figure 2A). Inputs enter the superficial layer where they are excitatory to dendrites and, in parallel, to feedforward inhibitory interneurons to the dendrites. PC axon branches give rise to internal feedback circuits that are widespread and excitatory to themselves and other PCs, and to interneurons that spread feedback and lateral inhibition. Output axons go to other parts of cortex or basal ganglia, and to the hypothalamus. These are IT cells. The only exception is a small projection from dorsal cortex to the superior colliculus, which may reflect dominance of the dorsal cortex by visual input (Fournier et al., 2015). These features are built into the simple circuit for all three types of three-layer cortex (Figure 2A). We term this PC with its feedforward and feedback excitatory and inhibitory circuits a basic circuit module for the cerebral cortex.

Olfactory cortex is expanded from this basic circuit module into sublayers related to each of the three PC subtypes (Figure 2B). Each subtype projects within the cortex, qualifying it as an IT cell, processing inputs (the multidimensional encoding of olfactory molecules) for further projection to higher processing (in orbitofrontal cortex) and eventually to motor output via entorhinal cortex. Association fibers are major constituents defining the laminae, and are subjects of current investigation.

How might the neocortex have evolved based on the basic circuit module? A simplified representation of the modules of multi-layer neocortex (Figure 2C) shows the IT cell. This appears to be the same type as in the three-layer cortex, interacting primarily with other forebrain regions at higher levels of associative function. In early amniotes, IT cells performed these higher functions almost entirely “offline” from the main flow of sensory input and motor outflow in subcortical brain regions. As with olfactory cortex, the higher associative functions of IT cells were inherited from their three-layer antecedents.

A major innovation in neocortex is the PT cell. Through this cell in layer 5b, its axon potentially connects to virtually all levels of the central nervous system. Kita and Kita (2012) have shown labeling of PT branches to other parts of ipsilateral cortex and basal ganglia, and to the thalamus and especially on down through the midbrain and hindbrain to the spinal cord. Individual neurons do not branch to all possible targets, but rather to subsets, which vary from neuron to neuron. The complexities of these multiple subcerebral connections may be as profound as complexities of the interactions within the expanded IT network of the neocortex itself.

The new principle of the neocortex is therefore not the associative network of IT cells *per se*, but two things: first, elaboration of the associative networks inherited from three layer cortex to give higher functions that include not only multidimensional perception and memory but also planning and execution; and second, connections through PT cells to give the cortex direct influence on the entire neuraxis. Far from being a level of higher associative function “offline” from the lower centers, the neocortex makes its higher functions available for direct insertion at all levels into the ongoing interactions of the neuraxis with the environment. Increased layering of IT cells, and increased elaboration of many cortical areas based on different sensory and motor connectivity, give neocortex its immense power in representing the world and acting upon it.

The CT PC in layer 6 connects specifically to the thalamic nuclei and thalamic reticular nucleus. It plays several roles in modulating vastly increased sensory input that occurs in the neocortex depending on motor output in the context of the behavioral state (Thomson, 2010). The fact that the CT and PT cells are found in specific layers suggests that they are inserted into the main multilayer framework of the IT cells. We speculate that CT cells evolved in their positions to direct output from IT cells to the thalamus, and that PT cells were placed to collect IT outputs to distribute throughout the neuraxis.

All three neocortical cell types have basic circuit modules of a principle neuron and its interneurons similar to their three layer counterparts, showing the conservation of principle. Given this basic module format, in each area are specific adaptations of cell morphology, subtypes and connectivities.

Other changes in mammals involved increases in information from somatosensation, motor control and in certain clades from audition and vision. This multidimensional inflow evidently required staging to put the information into a common form readily processed by the different cortical circuits by excitatory TC inputs. There are several types of TC projections (Clascá et al., 2012); two important types are “core” and “matrix”. Prominent in sensory systems is the “core” type of thalamic nuclei, with axons that target layer 4, where they activate pyramidal, stellate and other specialized IT cells (Figure 2B). The TC → layer 4 connection is present in specialized sensory systems such as rodent barrel cortex, and in “agranular” areas such as motor cortex, albeit in a diminutive, prototypical form (Yamawaki et al., 2014). The other major type of TC projection is the “matrix” type that targets layer 1 and layer 5A; this is probably the most prevalent type in the neocortex and evolutionarily the oldest. Layers may thus provide separation of targets for intralaminar interactions.

Not shown in Figure 2 is the evolution in neocortex of interneurons, especially the three types expressing Sst, Vip, and Pvalb, each with its specific morphology, local hodology and control of different integrated modules of principal neurons.

## Olfaction as A Driver of Neocortical Evolution

We can now see neocortex as a complex microcircuit based on four excitatory cell classes distributed across multiple layers. The superficial layers receiving major inputs to layer 1, the presence of IT neurons in multiple layers, and wide cortico-cortical projections of IT cells resemble the organization of three-layer cortex (Luzzati, 2015). It is a reasonable hypothesis that IT pyramidal neurons of the neocortex originated from amniote three-layer cortex, but what factors drove this transformation?

Duplication of the olfactory receptor genome in the antecedents of mammals may have been a dominant driver of neocortical evolution (Rowe et al., 2011; Rowe and Shepherd, 2016; Rowe, 2017). Peripheral sensory arrays are known to influence central organization and, through epigenetic population matching, cortical re-organization and increased neuron numbers may have been driven by connectional invasions from peripheral sensory cell populations (Katz and Lasek, 1978; Krubitzer and Kaas, 2005). Fossils documenting mammalian antecedents record initial pulses of encephalization tied to expansion of olfactory cortex, and only later is there evidence suggesting neocortical differentiation. Increased input from teeth, hair and other peripheral systems were influential, but to a lesser degree.

Gene expression offers clues to developmental programs that may be specific for the cells in the regions involved. Luzzati (2015) found localization of Doublecortin (DCX+/Tbr+) in olfactory cortex and in regions of neocortex believed to be derived from the dorsal cortex, but not in dorsal cortex itself. This supported Reiner et al.’s (1998) proposition that the superficial layers are a mammalian novelty. However, sharing the same gene expressed in olfactory three-layer cortex and upper layer neocortex supported the possibility that the superficial layers of dorsal cortex were produced by co-option of the generation program for cell types in olfactory cortex (Luzzati, 2015; Klingler, 2017). Similarities between olfactory cortex and layers 2 and 3 also include *in situ* hybridization data from the Allen Mouse Brain Atlas of many shared expressed genes between these regions. Both regions have interhemispheric projections, olfactory cortex through the anterior commissure, which also carries neocortical projections in monotremes and marsupials (in placentals via the corpus callosum), and both olfactory cortex and neocortex project to the lateral entorhinal cortex to reach the hippocampus.

These similarities further support “a potential role for the olfactory system as a driver for the evolution of the neocortex” (Luzzati, 2015).

As Aboitiz and Montiel (2015) comment: “our hypothesis has common ground with those proposed by Lynch (1986), Rowe et al. (2011) and Rowe and Shepherd (2016) that olfactory systems were key in early mammalian evolution. Here we add to these hypotheses the role of the emergent isocortex as a multimodal interface in the olfactory-hippocampal axis for behavioral navigation”.

Layering in olfactory cortex may reflect expansion of the olfactory repertoire that evolved in early mammalian history. The long fibers of semilunar cells have remained within their layer (Wilson and Barkai, 2010; Susuki and Bekkers, 2012; Brunjes and Osterberg, 2015), implying that specific associational fiber systems in layers may be required by increased odor object processing with expanded odor input. As noted above, pyramidal neuron types at different depths develop in an inside-out sequence in olfactory cortex and in mammalian neocortex. The olfactory system thus appeared to play a key role in neocortical evolution, via epigenetic effects of odorant receptor gene duplication and possibly by co-opting a genetic module originally expressed in three-layer olfactory cortex to produce the six IT layers of the neocortex.

In summary, an evolutionary context for hodologically-defined cell types provides a new framework for understanding neocortical lamination. IT cells of dorsal and olfactory three-layer cortex appear to have higher associative functions that provided the basis for IT cells with greatly increased interconnectivity in mammalian neocortex. The six-layer neocortex manifests evolution of increased IT connectivity, increased cell populations, and expanded interlaminar integration underlying columnar organization. Current studies now aim to elucidate specific thalamic inputs into L4IT cells for preprocessing, specific integration with thalamus through CT cells, and the final common path through PT cells to allow higher associative functions generated by IT cells to have direct control over much of the neuraxis.

## Author Contributions

GMS originated the manuscript based on an ongoing collaboration with TBR, wrote the first draft, combined the comments from TBR, and finalized it for submission. TBR read the first draft, contributed critical input on the evolutionary context for the perspective, and reviewed for submission.

## Conflict of Interest Statement

The authors declare that the research was conducted in the absence of any commercial or financial relationships that could be construed as a potential conflict of interest.

## References

[B1] Aboitiz F. Montiel J. F. (2015). Olfaction, navigation, and the origin of isocortex. Front. Neurosci. 9:402. 10.3389/fnins.2015.00402 26578863 PMC4621927

[B3] Brunjes P. C. Osterberg S. K. (2015). Developmental markers expressed in neocortical layers are differentially exhibited in olfactory cortex. PLoS One 10:e0138541. 10.1371/journal.pone.0138541 26407299 PMC4583488

[B4] Clascá F. Rubio-Garrido P. Jabaudon D. (2012). Unveiling the diversity of thalamocortical neuron subtypes. Eur. J. Neurosci. 35, 1524–1532. 10.1111/j.1460-9568.2012.08033.x 22606998

[B5] Diodato A. Ruinart de Brimont M. Yim Y. S. Derian N. Perrin S. Pouch J.. (2016). Molecular signatures of neural connectivity in the olfactory cortex. Nat. Commun. 7:12238. 10.1038/ncomms12238 27426965 PMC4960301

[B6] Fournier J. Müller C. M. Laurent G. (2015). Looking for the roots of cortical sensory computation in three-layer cortices. Curr. Opin. Neurobiol. 31, 119–126. 10.1016/j.conb.2014.09.006 25291080 PMC4898590

[B7] Güntürkün O. Stacho M. Ströckens F. (2017). “The brains of reptiles and birds,” in Evolution of Nervous Systems 2, (Vol. 1) ed. Kaas J. (Elsevier: Academic Press), 171–221.

[B9] Haberly L. B. (1985). Neuronal circuitry in olfactory cortex: anatomy and functional implications. Chem. Senses. 10, 219–238. 10.1093/chemse/10.2.219

[B8] Haberly L. B. Shepherd G. M. (1973). Current-density analysis of summed evoked potentials in opossum prepyriform cortex. J. Neurophysiol. 36, 789–802. 4713320 10.1152/jn.1973.36.4.789

[B10] Harris K. D. Shepherd G. M. G. (2015). The neocortical circuit: themes and variations. Nat. Neurosci. 18, 170–181. 10.1038/nn.3917 25622573 PMC4889215

[B11] Katz M. J. Lasek R. J. (1978). Evolution of the nervous system: role of ontogenetic mechanisms in the evolution of matching populations. Proc. Natl. Acad. Sci. U S A 75, 1349–1352. 10.1073/pnas.75.3.1349 274722 PMC411468

[B12] Kita T. Kita H. (2012). The subthalamic nucleus is one of multiple innervation sites for long-range corticofugal axons: a sigle-axon tracing study in the rat. J. Neurosci. 32, 5990–5999. 10.1523/JNEUROSCI.5717-11.2012 22539859 PMC3479642

[B13] Klingler E. (2017). Development and organization of the evolutionarily conserved three-layered olfactory cortex. eNeuro 4:ENEURO.0193-16.2016. 10.1523/ENEURO.0193-16.2016 28144624 PMC5272922

[B14] Kriegstein A. R. Connors B. W. (1986). Cellular physiology of the turtle visual cortex: synaptic properties and intrinsic circuitry. J. Neurosci. 6, 178–191. 2868076 10.1523/JNEUROSCI.06-01-00178.1986 PMC6568623

[B15] Krubitzer L. Kaas J. (2005). The evolution of the neocortex in mammals: how is phenotypic diversity generated? Curr. Opin. Neurobiol. 15, 444–453. 10.1016/j.conb.2005.07.003 16026978

[B16] Luzzati F. (2015). A hypothesis for the evolution of the upper layers of the neocortex through co-option of the olfactory cortex developmental program. Front. Neurosci. 9:162. 10.3389/fnins.2015.00162 26029038 PMC4429232

[B17] Lynch G. (1986). Synapses, Circuits, and the Beginnings of Memory. Cambridge, MA: MIT Press.

[B18] Naumann R. K. Laurent G. (2017). “Function and evolution of the reptilian cerebral cortex,” in Evolution of Nervous Systems 2, (Vol. 1) ed. Kaas J. (Elsevier: Academic Press), 491–518.

[B19] Naumann R. K. Ondracek J. M. Reiter S. Shein-Idelson M. Tosches M. A. Yamawaki T. M.. (2015). The reptilian brain. Curr. Biol. 25, R317–R321. 10.1016/j.cub.2015.02.049 25898097 PMC4406946

[B20] Neville K. R. Haberly L. B. (2004). “Olfactory cortex,” in The synaptic Organization of the Brain, ed. Shepherd G. M. (New York, NY: Oxford University Press), 415–454.

[B21] Oberlaender M. de Kock D. P. J. Bruno R. M. Ramirez A. Meyer H. S. Dercksen V. J.. (2012). Cell type-specific three-dimensional structure of thalamocortical circuits in a column of rat vibrissal cortex. Cereb. Cortex 22, 2375–2391. 10.1093/cercor/bhr317 22089425 PMC3432239

[B22] Reiner A. Medina L. Veenman C. L. (1998). Structural and functional evolution of the basal ganglia in vertebrates. Brain Res. Rev. 28, 235–285. 10.1016/s0165-0173(98)00016-2 9858740

[B24] Rowe T. B. Macrini T. E. Luo Z.-X. (2011). Fossil evidence on origin of the mammalian brain. Science 332, 955–957. 10.1126/science.1203117 21596988

[B25] Rowe T. B. (2017). “The emergence of mammals,” in Evolution of Nervous Systems 2, (Vol. 2) ed. Kaas J. (Oxford: Elsevier), 1–52.

[B23] Rowe T. B. Shepherd G. M. (2016). The role of ortho-retronasal olfaction in mammalian cortical evolution. J. Comp. Neurol. 524, 471–495. 10.1002/cne.23802 25975561 PMC4898483

[B26] Shepherd G. M. (1974). The Synaptic Organization of the Brain. New York, NY: Oxford University Press.

[B27] Shepherd G. M. (1988). Neurobiology. 2nd Edn. New York, NY: Oxford University Press.

[B28] Shepherd G. M. (2011). The microcircuit concept applied to cortical evolution: from three-layer to six-layer cortex. Front. Neuroanat. 5:30. 10.3389/fnana.2011.00030 21647397 PMC3102215

[B29] Smith L. M. Ebner F. F. Colonnier M. (1980). The thalamocortical projection in Pseudemys turtles: a quantitative electron microscopic study. J. Comp. Neurol. 190, 445–461. 10.1002/cne.901900304 7391267

[B30] Streidter G. F. (2005). Principles of Brain Evolution. Sunderland, MA: Sinauer Associates Inc.

[B2] Susuki N. Bekkers J. M. (2012). Two layers of synaptic processing by principal neurons in piriform cortex. J. Neurosci. 31, 2156–2166. 10.1523/JNEUROSCI.5430-10.2011 21307252 PMC6633060

[B31] ten Donkelaar H. J. (1998). “Reptiles,” in The Central Nervous System of Vertebrates, (Vol. 2) eds Nieuwenhuys R. ten Donkelaar H. J. Nicholson C. (Berlin: Springer), 1315–1524.

[B32] Thomson A. (2010). Neocortical layer 6, a review. Front. Neuroanat. 4:13. 10.3389/fnana.2010.00013 20556241 PMC2885865

[B33] Ulinski P. S. (1983). Dorsal Ventricular Ridge; A Treatise on Forebrain Organization in Reptiles and Birds. New York, NY: John Wiley and Sons.

[B34] Wilson D. A. Barkai E. (2010). “Olfactory cortex,” in Handbook of Brain Microcircuits, 2nd Edn. eds Shepherd G. M. Grillner S. (New York, NY: Oxford University Press), 263–276.

[B35] Yamawaki N. Borges K. Suter B. A. Harris K. D. Shepherd G. M. G. (2014). A genuine layer 4 in motor cortex with prototypical synaptic circuit connectivity. Elife 3:e05422. 10.7554/eLife.05422 25525751 PMC4290446

